# ***α***-Lipoic Acid Inhibits *Helicobacter pylori*-Induced Oncogene Expression and Hyperproliferation by Suppressing the Activation of NADPH Oxidase in Gastric Epithelial Cells

**DOI:** 10.1155/2014/380830

**Published:** 2014-08-19

**Authors:** Eunyoung Byun, Joo Weon Lim, Jung Mogg Kim, Hyeyoung Kim

**Affiliations:** ^1^Department of Food and Nutrition, Brain Korea 21 PLUS Project, College of Human Ecology, Yonsei University, Seoul 120-749, Republic of Korea; ^2^Department of Microbiology, Hanyang University College of Medicine, Seoul 133-791, Republic of Korea

## Abstract

Hyperproliferation and oncogene expression are observed in the mucosa of *Helicobacter pylori- (H. pylori-)* infected patients with gastritis or adenocarcinoma. Expression of oncogenes such as *β*-catenin and c-myc is related to oxidative stress. *α*-Lipoic acid (*α*-LA), a naturally occurring thiol compound, acts as an antioxidant and has an anticancer effect. The aim of this study is to investigate the effect of *α*-LA on *H. pylori*-induced hyperproliferation and oncogene expression in gastric epithelial AGS cells by determining cell proliferation (viable cell numbers, thymidine incorporation), levels of reactive oxygen species (ROS), NADPH oxidase activation (enzyme activity, subcellular levels of NADPH oxidase subunits), activation of redox-sensitive transcription factors (NF-*κ*B, AP-1), expression of oncogenes (*β*-catenin, c-myc), and nuclear localization of *β*-catenin. Furthermore, we examined whether NADPH oxidase mediates oncogene expression and hyperproliferation in *H. pylori*-infected AGS cells using treatment of diphenyleneiodonium (DPI), an inhibitor of NADPH oxidase. As a result, *α*-LA inhibited the activation of NADPH oxidase and, thus, reduced ROS production, resulting in inhibition on activation of NF-*κ*B and AP-1, induction of oncogenes, nuclear translocation of *β*-catenin, and hyperproliferation in *H. pylori*-infected AGS cells. DPI inhibited *H. pylori*-induced activation of NF-*κ*B and AP-1, oncogene expression and hyperproliferation by reducing ROS levels in AGS cells. In conclusion, we propose that inhibiting NADPH oxidase by *α*-LA could prevent oncogene expression and hyperproliferation occurring in *H. pylori*-infected gastric epithelial cells.

## 1. Introduction

Epidemiologic studies showed that* Helicobacter pylori* (*H. pylori*) infection increased the incidence of gastric cancer up to 6-fold [1–4]. The key features of developing gastric cancer are hyperproliferation and oncogene expression of gastric epithelial cells. Oncogenes such as *β*-catenin and c-myc stimulate cell proliferation and promote malignant changes.* H. pylori* infection is associated with hyperproliferation of gastric epithelial cells in humans and experimental animals [5–7]. Nuclear level of *β*-catenin was increased by* H. pylori* infection in gastric epithelial cells [6, 7]. However, the mechanisms by which* H. pylori* infection promotes epithelial hyperproliferation remain poorly understood.

*β*-catenin has a key role in inflammation and cancer development [8]. Cytosolic and nuclear levels of *β*-catenin are tightly regulated by signaling molecules in the cells [9]. Upon activation, *β*-catenin is stabilized and translocated into the nucleus. In the nucleus, *β*-catenin binds to TCF family and serves as a transcriptional regulator [10, 11]. Recent study showed that* H. pylori* infection stimulates release of *β*-catenin which in turn is translocated into nucleus [12]. Nuclear translocation of *β*-catenin and cell proliferation have been related to c-myc in* H*.* pylori*-infected cells [13]. c-myc is one of the genes regulated by *β*-catenin, whose expression is directly activated by *β*-catenin/TCF in colon cancers [14]. As a protooncogene, c-myc stimulates the expression of target genes, which plays important roles in uncontrolled cell proliferation by binding to consensus 5′-CACGTG-3′ nucleotide sequences in the region of these genes and behaving as a transcriptional activator [15].

Previously, we found that reactive oxygen species (ROS) were produced to induce IL-8 expression in* H. pylori*-infected gastric epithelial cells, which may contribute to neutrophil recruitment to the infected tissues [16]. Several studies suggest that nicotinamide adenine dinucleotide phosphate (NADPH) oxidase is involved in ROS production of* H. pylori*-infected gastric mucosa in humans and mice [17–19]. For the activation of NADPH oxidase, the assembly of membrane-integrated cytochrome b558 (a heterodimer formed by gp91^phox^ and p22^phox^) and cytosolic components p47^phox^, p67^phox^, and GTPase Rac are necessary. Electron transfer occurs from NADPH to molecular O_2_^−^ which is spontaneously converted to H_2_O_2_ [20]. Therefore, membrane translocation of cytosolic subunits is a main switch in NADPH oxidase activation.

Nollet et al. [21] reported that there are binding sites for redox-sensitive transcription factors NF-*κ*B and AP-1in the promoter region of *β*-catenin. NF-*κ*B, AP-1, and *β*-catenin signaling contributed to survival of TNF-*α*-treated hepatocytes* in vitro* [22]. Since NF-*κ*B and AP-1 are activated by ROS, NADPH oxidase-generated ROS may induce expression of oncogenes (*β*-catenin, c-myc) by activating NF-*κ*B and AP-1 in* H. pylori*-infected gastric epithelial cells.

*α*-Lipoic acid (*α*-LA), also known as thioctic acid, is a naturally occurring antioxidant that is synthesized in small amounts in plants and animals including humans [23]. *α*-LA is considered as an ideal antioxidant since it possesses many beneficial characteristics, including free-radical quenching activity, recycling other antioxidants, and suppressive effects on redox-sensitive gene expression [24]. *α*-LA inhibited NF-*κ*B activation and protected oxidative cell injury [25]. Growth inhibitory effect of *α*-LA was shown in ovarian epithelial cancer cells [26]. Wenzel et al. [27] demonstrated that *α*-LA has carcinostatic effects in cancer patients by increasing glutathione and reducing oxidative stress in cancer cells.

The purpose of the present study is to investigate the effect of *α*-LA on hyperproliferation and oncogene expression in* H. pylori*-infected gastric epithelial cells by determining cell proliferation (viable cell numbers, thymidine incorporation), ROS levels, NADPH oxidase activation (enzyme activity, subcellular levels of NADPH oxidase subunits), activation of redox-sensitive transcription factors (NF-*κ*B, AP-1), expression of oncogenes (*β*-catenin, c-myc), and nuclear localization of *β*-catenin. Furthermore, we examined the effect of diphenyleneiodonium (DPI), an inhibitor of NADPH oxidase, on oncogene expression and hyperproliferation in* H. pylori*-infected AGS cells to elucidate whether NADPH oxidase mediates* H. pylori*-induced proliferation.

## 2. Materials and Methods

### 2.1. Bacterial Strain

*H. pylori*, strain NCTC 11637, was obtained from the American Type Culture Collection (Rockville, MD). The genotype of this bacterium is* cagA* ^*+*^ *and vacA* ^*+*^ and was inoculated on chocolate agar plates (Becton Dickinson Microbiology Systems, Cockeysville, MD, USA) at 37°C under microaerophilic conditions using an anaerobic chamber (BBL Campy Pouch System, Becton Dickinson Microbiology Systems) [28].

### 2.2. Cell Line and Culture* H. pylori* Infection

A human gastric epithelial cell line AGS (gastric adenocarcinoma, ATCC CRL 1739) was purchased from the American Type Culture Collection. The cells were grown in complete medium, consisting of RPMI 1640 medium supplemented with 10% fetal bovine serum, 2 mM glutamine, 100 U/mL penicillin, and 100 *μ*g/mL streptomycin (Sigma, St. Louis, MO, USA) [28]. AGS cells were seeded and cultured overnight to reach 80% confluency. Before* H. pylori* infection, the cells were washed with antibiotic-free culture medium. Whole* H. pylori* was harvested and suspended in antibiotic-free RPMI 1640 medium supplemented with 10% fetal bovine serum and treated to AGS cells.

### 2.3. Experimental Protocol

Prior to the experiment on *α*-LA, the cells were cultured at bacterium/cell ratio of 10 : 1, 20 : 1, and 50 : 1 to determine the appropriate density of* H. pylori* to the cells for cell proliferation, thymidine incorporation (at 8 h), oncogene expression (at 24 h), ROS production (at 30 min), and NADPH oxidase activity (at 30 min). At bacterium/cell ratio of 50 : 1, cells were cultured for 24 h to determine oncogene expression at mRNA and protein levels. For the effect of *α*-LA or DPI, the cells were cultured at bacterium/cell ratio of 50 : 1. *α*-LA and DPI were purchased from Sigma-Aldrich (St. Louis, MO, USA). *α*-LA was dissolved in ethanol while DPI was dissolved in dimethylsulfoxide (DMSO). The cells were pretreated with *α*-LA (10 *μ*M, 20 *μ*M) or DPI (1 *μ*M, 2 *μ*M) for 2 h before* H. pylori* infection. Cell proliferation, thymidine incorporation (at 8 h), oncogene expression (at 24 h), ROS production (at 30 min), activity and cellular localization of NADPH oxidase (at 30 min), and activation of NF-*κ*B and AP-1 (at 1 h) were determined. The control group received ethanol or DMSO instead of *α*-LA or DPI. Total volume of ethanol or DMSO treated to AGS cells was less than 0.5%.

### 2.4. Cell Proliferation

Cell proliferation was determined by viable cell numbers. Cell numbers were determined by direct counting with a hemocytometer using a trypan blue exclusion test (0.2% trypan blue).

### 2.5. [^3^ *H*] Thymidine Incorporation

AGS cells (1 × 10^4^/well) were pretreated with *α*-LA for 2 h and cultured in the presence or absence of* H. pylori *in a 24-well culture plate. After 24 h-culture, 1 *μ*Ci/mL [^3^H] thymidine (Amersham Biosciences) was added to the cells, and the cells were cultured for an additional 8 h. The cells were then washed twice with phosphate-buffered saline, incubated in 10% trichloroacetic acid for 30 min, and incubated with a solution consisting of 0.3 M NaOH and 1% SDS for 1 h. The cells were extracted with vortexing and the radioactivity was determined in a Packard liquid scintillation counter (Packard Instrument Co. Inc., Grove, IL, USA). The relative amount of [^3^H] thymidine incorporation, which reflects the extent of DNA synthesis, is expressed as a percentage of that shown in the cells cultured in the absence of* H. pylori*. The amount of [^3^H] thymidine incorporation of the cells cultured in the absence of* H. pylori* is considered as 100% [29].

### 2.6. Real-Time PCR Analysis for *β*-Catenin and c-myc

Total RNA was isolated by TRI reagent (RNA/DNA/Protein isolation reagent, Molecular Research Center, Inc., Cincinnati, OH, USA). Total RNA was converted into cDNA by reverse transcription process using a random hexamer and M-MLV reverse transcriptase (Promega, Madison, WI, USA) with conditions at 23°C for 10 min, 37°C for 60 min, and 95°C for 5 min. The cDNA was used for real-time PCR with specific primers for *β*-catenin, c-myc, and *β*-actin. Sequences of *β*-catenin primers were 5′-GTTCGTGCACATCAGGATAC-3′ (forward primer) and 5′-CGATAGCTAGGATCATCCTG-3′ (reverse primer), giving a 529 bp PCR product. Sequences of c-myc primers were 5′-GGACGACGAGACCTTCATCAA-3′ (forward primer) and 5′-CCAGCTTCTCTGAGACGAGCTT-3′ (reverse primer), giving a 92 bp PCR product. For *β*-actin, the forward primer was 5′-ACCAACTGGGACGACATGGAG-3′ and the reverse primer was 5′-GTGAGGATCTTCATGAGGTAGTC-3′, giving a 353 bp PCR product. For PCR amplification, the cDNA was amplified by 40 cycles, denaturation at 95°C for 15 sec, annealing at 60°C for 15 sec, and extension at 72°C for 45 sec. *β*-Actin gene was amplified in the same reaction to serve as the reference gene.

### 2.7. Western Blot Analysis

Whole cell extracts, membrane extracts, cytosolic extracts, and nuclear extracts were prepared as described previously [28]. 100–200 *μ*g of protein was loaded per lane, separated by 8–12% SDS-polyacrylamide gel electrophoresis under reducing conditions, and transferred onto nitrocellulose membranes (Amersham, Inc., Arlington Heights, IL, USA) by electroblotting. The transfer of protein was verified using reversible staining with Ponceau S. Membranes which were blocked using 3% nonfat dry milk. The proteins were detected with antibodies for *β*-catenin, c-myc, p47, p67, NOX-1, aldolase A, histone H1, and actin (all from Santa Cruz Biotechnology) dilution in TBS-T containing 3% dry milk, and incubated overnight at 4°C, followed by secondary antibodies (anti-goat, anti-mouse, or anti-rabbit) conjugated to horseradish peroxidase and determination of enhanced chemiluminescence (Amersham) using exposure to BioMax MR film (Kodak, Rochester, NY) [30].

### 2.8. Measurement of ROS Levels

After 30 min of* H. pylori* infection, the cells were loaded with 10 *μ*M dichlorofluorescein diacetate (DCF-DA; Molecular Probes, Eugene, OR, USA) for 30 min, washed, and scraped off into 1 mL of PBS. The DCF fluorescence was measured (excitation at 495 nm and emission at 535 nm) with a Vctior3 multilabel counter (PerkinElmer Life and Analytical Sciences, Boston, MA, USA). ROS trapped into the cells were expressed as therelative increase [31].

### 2.9. Electrophoretic Mobility Shift Assay (EMSA)

A NF-*κ*B gel shift oligonucleotide (AGTTGAGGGGACTTTCCCAGGC) and a AP-1 gel-shift oligonucleotide (CGCTTGATA GTCAGCCGGAA) (all from Promega, Madison, WI, USA) were labeled with [^32^P] dATP (Amersham) using the T4 polynucleotide kinase (GIBCO, Grand Island, NY, USA). The end-labeled probe was purified from an unincorporated [^32^P] dATP using a Bio-Rad purification column (Bio-Rad Laboratories) and recovered in Tris-EDTA buffer (TE). Nuclear extracts (3 *μ*g) were incubated with the buffer containing ^32^P-labeled NF-*κ*B or AP-1 consensus oligonucleotide for 30 min and subjected to electrophoretic separation on a nondenaturing acrylamide gel. The gels were dried at 80°C for 2 h and exposed to a radiography film for 6–18 h at −70°C with intensifying screens [28].

### 2.10. Determination of NADPH Oxidase Activity

The assay was performed in 50 mM Tris-Mes buffer, pH 7.0, containing 2 mM KCN, 10 *μ*M lucigenin and 100 *μ*M NADPH as the substrate. The reaction was started by addition of membrane extracts containing 10 *μ*g protein. The photon emission was measured every 15 sec for 5 min in a microtiterplate luminometer (Micro-Lumat LB 96 V luminometer, Berthold, NH, USA). NADPH oxidase activity was also monitored by addition of cytosolic extracts to the reaction mixture as a negative control [32].

### 2.11. Immunofluorescence Staining for *β*-Catenin

The cells were cultured in the presence or absence of* H. pylori* for 24 h on Lab-TeK chamber slide glasses and fixed with cold 100% methanol. The fixed cells were blocked for 30 min in a blocking solution and then incubated for 1 h with primary antibody for *β*-catenin. After washing with PBS, the cells were reacted with FITC-labeled goat anti-mouse IgG antibody for 1 h. After removal of the secondary antibodies, the cells were washed with PBS and covered with the antifade medium Vectashield containing 4′,6-diamidino-2-phenylindole (DAPI). The preparations were stored for 1 h to allow saturation with DAPI. The cells stained with FITC-labeled antibody for *β*-catenin were examined with a laser-scanning confocal microscope (LSM510, Carl Zeiss Inc., Oberkochen, Germany) [33].

### 2.12. Statistical Analysis

Results are expressed as mean ± S.E.M. of four separate experiments. Analysis of variance (ANOVA), followed by Newman-Keul's post hoc test was used for statistical analysis. *P* < 0.05 was considered statistically significant.

## 3. Results

### 3.1. *H. pylori* Induces Hyperproliferation and Expression of *β*-Catenin and c-myc in AGS Cells

**Figure 1:**
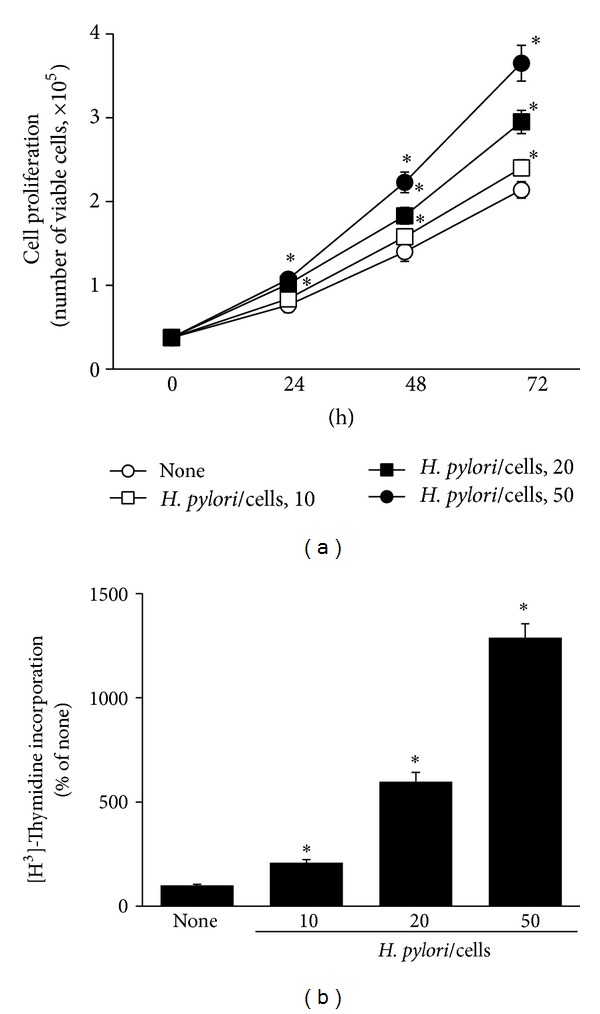
Cell proliferation of AGS cells infected with* H. pylori. *AGS cells were cultured in the presence or absence of* H. pylori *(at bacterium/cell ratio of 10, 20, 50 : 1). Cell proliferation was assessed by viable cell numbers and [^3^H] thymidine incorporation. (a) Viable cell numbers were determined by trypan blue exclusion assay at the indicated time period. (b) The cells were treated with [^3^H] thymidine at 24 h after* H. pylori* infection and incubated for 8 h. The amount of [^3^H] thymidine incorporation of AGS cells cultured in the absence of* H. pylori* is considered as 100%. _ _*P < 0.05 versus corresponding none (the cells cultured in the absence of* H. pylori*).

**Figure 2:**
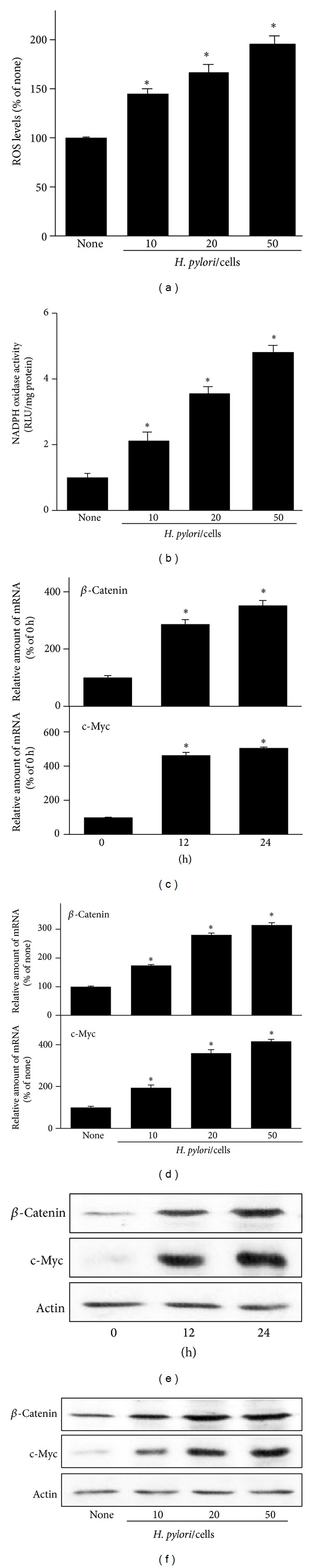
ROS levels, NADPH oxidase activity, and mRNA and protein levels of *β*-catenin and c-myc in AGS cells infected with* H. pylori. *(a, b, d, f) The cells were cultured in the presence or absence of* H. pylori *(at bacterium/cell ratio of 10, 20, 50 : 1) for 24 h. (c, e) AGS cells were cultured in the presence or absence of* H. pylori *(bacterium/cell ratio of 50 : 1) for the indicated time period. (a) ROS levels were determined by DCF fluorescence after 30 min of* H. pylori* infection. The levels of ROS trapped in the cells and cultured in the absence of* H. pylori* (none) are considered as 100%. (b) NADPH oxidase activity was determined by lucigenin assay using membrane extracts of the cells. NADPH oxidase activity was also monitored by addition of cytosolic extracts to the reaction mixture as a negative control. (c–f) mRNA and protein expression were determined by real-time PCR and Western blot analysis, respectively. Actin served as a loading control. _ _*P < 0.05 versus 0 h or none (the cells cultured in the absence of* H. pylori*).

During 72 h-culture,* H. pylori* increased cell numbers as compared to the cells without infection (Figure 1(a)). With cell proliferation,* H. pylori* elicited an increase in thymidine incorporation, an index of DNA synthesis at 24 h-culture (Figure 1(b)).* H. pylori*-induced proliferation, activation of NADPH oxidase, ROS production, and oncogene expression were highest at bacterium/cell ratio of 50 : 1 as compared to those at 20 : 1 and 10 : 1 (Figures 1(b), 2(a), 2(b), 2(d), and 2(f)). The expression levels of *β*-catenin and c-myc were increased by* H. pylori* infection with culture time at bacterium/cell ratio of 50 : 1 (Figures 2(c) and 2(e)).

### 3.2. *α*-LA Suppresses* H. pylori*-Induced Hyperproliferation, Increase in ROS Levels, and NADPH Oxidase Activation in AGS Cells

**Figure 3:**
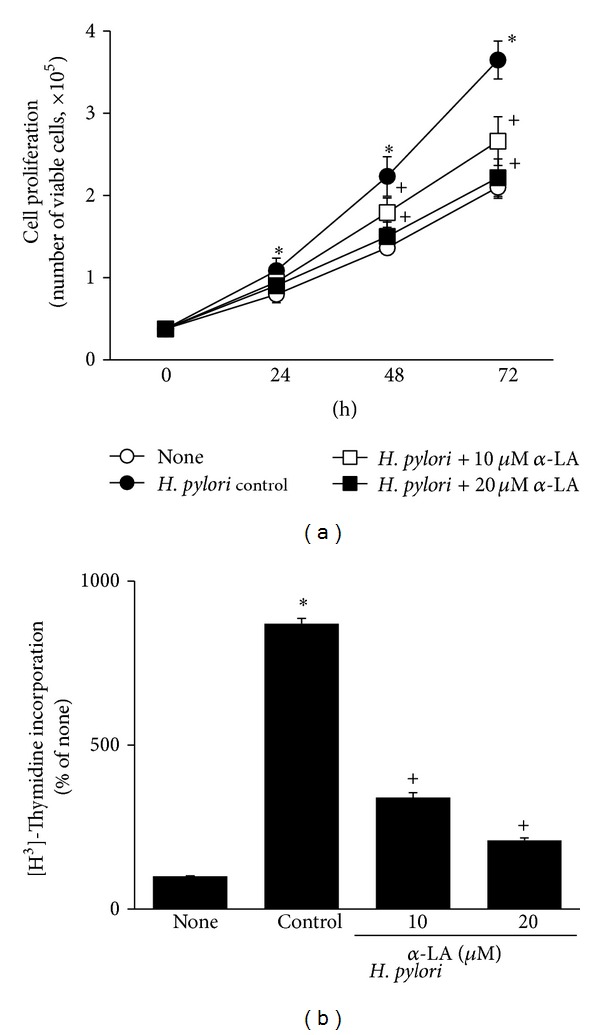
Effect of *α*-LA on cell proliferation of* H. pylori-*infected AGS cells. The cells were pretreated with *α*-LA for 2 h and cultured in the presence or absence of* H. pylori*. Cell proliferation was assessed by viable cell numbers and [^3^H] thymidine incorporation. (a) Viable cell numbers were determined by trypan blue exclusion assay for the indicated time period. (b) The cells were treated with [^3^H] thymidine at 24 h after* H. pylori* infection and incubated for 8 h. The amount of [^3^H] thymidine incorporation of AGS cells cultured in the absence of* H. pylori* is considered as 100%. _ _*P < 0.05 versus corresponding none (the cells cultured in the absence of* H. pylori*); ^+^ *P* < 0.05 versus corresponding* H. pylori* control (the cells cultured in the presence of* H. pylori* and treated without *α*-LA).

**Figure 4:**
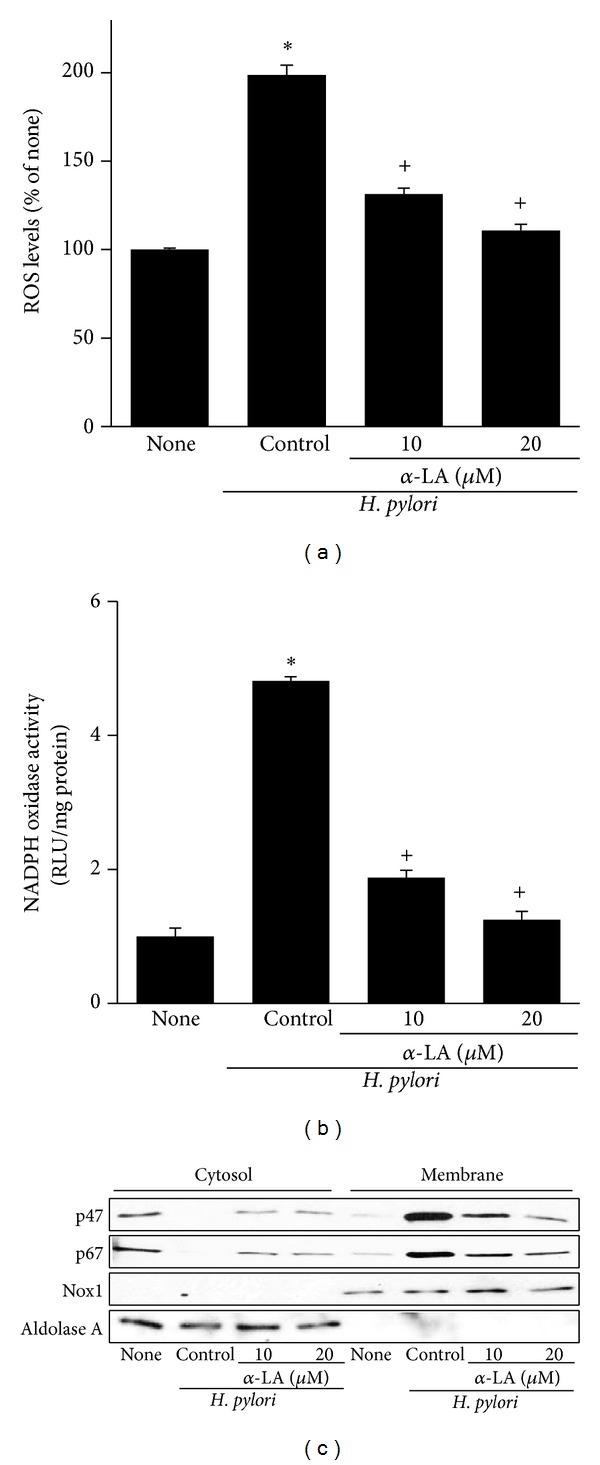
Effect of *α*-LA on ROS levels, NADPH oxidase activity, and levels of NADPH oxidase subunits in* H. pylori-*infected AGS cells. The cells were pretreated with *α*-LA for 2 h and cultured in the presence or absence of* H. pylori.* (a) ROS levels were determined by DCF fluorescence after 30 min of* H. pylori* infection. The levels of ROS trapped in the cells treated without DPI and cultured in the absence of* H. pylori* are considered as 100%. (b) NADPH oxidase activity was determined by lucigenin assay using membrane extracts of the cells. NADPH oxidase activity was also monitored by addition of cytosolic extracts to the reaction mixture as a negative control. (c), Protein levels of NADPH oxidase subunits (p47, p67) in cytosolic and membrane extracts were determined by Western blotting. Aldolase A and Nox1 were used as indices for cytosol and membrane, respectively. _ _*P < 0.05 versus none (the cells cultured in the absence of* H. pylori*); ^+^ *P* < 0.05 versus control (the cells cultured in the presence of* H. pylori* and treated without *α*-LA).

*α*-LA inhibited* H. pylori*-induced cell proliferation (determined by viable cell numbers during 72 h culture) and DNA synthesis (at 8 h culture) in AGS cells (Figures 3(a) and 3(b)). As shown in Figure 4(a),* H. pylori*-induced increase in ROS levels was reduced by *α*-LA treatment. Inhibitory effect of *α*-LA on* H. pylori*-induced cell proliferation and ROS production was higher at 20 *μ*M than 10 *μ*M (Figures 3 and 4(a)).

To further ensure the effect of *α*-LA on* H. pylori*-induced activation of NADPH oxidase, enzyme activity and subcellular levels of NADPH oxidase subunits were determined by lucigenin assay and Western blot analysis at 30 min culture (Figures 4(b) and 4(c)).* H. pylori-*induced increase in NADPH oxidase activity was suppressed by *α*-LA. For NADPH oxidase activation, the translocation of cytosolic subunits p47^phox^ and p67^phox^ to the membrane is required. As shown in Figure 4(c),* H. pylori*-induced translocation of cytosolic subunits p47^phox^ and p67^phox^ to membrane was inhibited by *α*-LA in AGS cells. Aldolase A and Nox1 as indices for cytosol and membrane, were not changed by* H. pylori* infection or treatment of *α*-LA.

### 3.3. *α*-LA Suppresses* H. pylori*-Induced Expression of Oncogenes, Activation of NF-*κ*B and AP-1, and Nuclear Translocation of *β*-Catenin in AGS Cells

**Figure 5:**
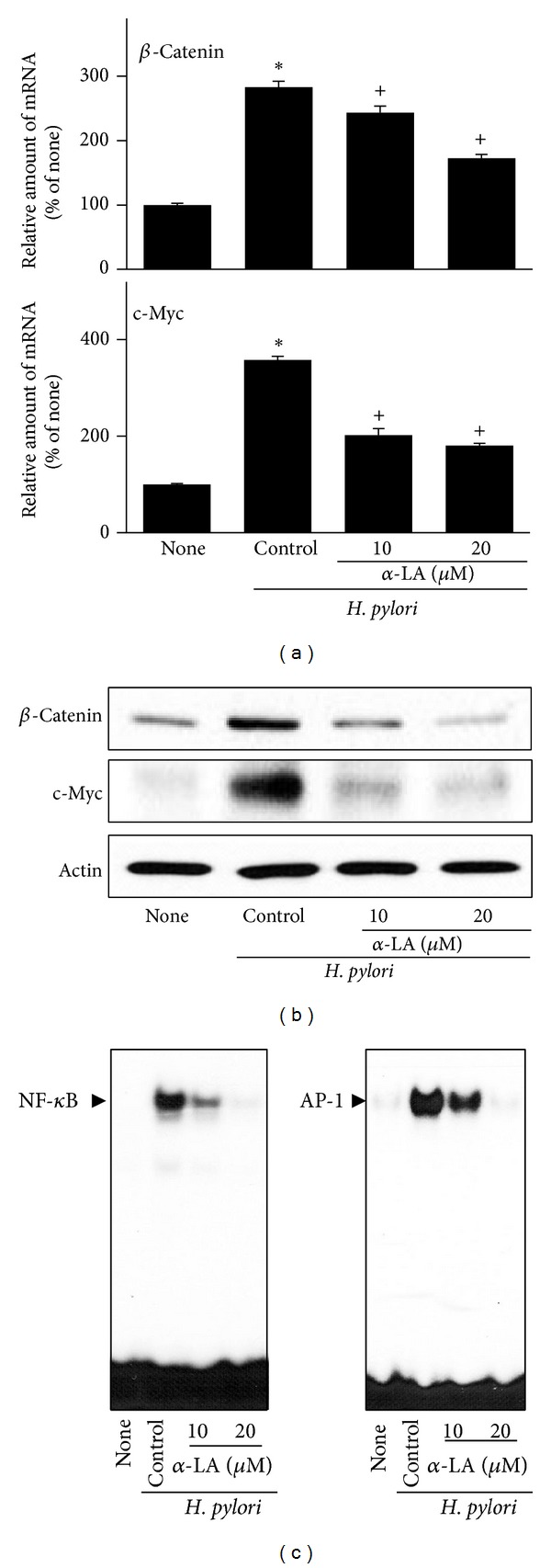
Effect of *α*-LA on expression of *β*-catenin and c-myc as well as activation of NF-*κ*B and AP-1 in* H. pylori-*infected AGS cells. The cells were pretreated with *α*-LA for 2 h and cultured in the presence or absence of* H. pylori *for 24 h (mRNA, protein levels) or 1 h (activation of NF-*κ*B and AP-1). (a) mRNA expression of *β*-catenin and c-myc was measured by real-time PCR analysis. (b) Protein levels of *β*-catenin and c-myc were determined by Western blot analysis. Actin served as a loading control. (c) EMSA was performed for DNA binding activities of NF-*κ*B and AP-1. _ _*P < 0.05 versus none (the cells cultured in the absence of* H. pylori*); ^+^ *P* < 0.05 versus control (the cells cultured in the presence of* H. pylori* and treated without *α*-LA).

*α*-LA inhibited* H. pylori*-induced expressions of *β*-catenin and c-myc in AGS cells at 24 h culture (Figures 5(a) and 5(b)). Furthermore, *α*-LA showed an inhibitory effect on* H. pylori*-induced activation of NF-*κ*B and AP-1 at 1 h culture (Figure 5(c)). Inhibitory effect of *α*-LA on* H. pylori*-induced oncogene expression and activation of NF-*κ*B and AP-1 was higher at 20 *μ*M than 10 *μ*M of *α*-LA.

**Figure 6:**
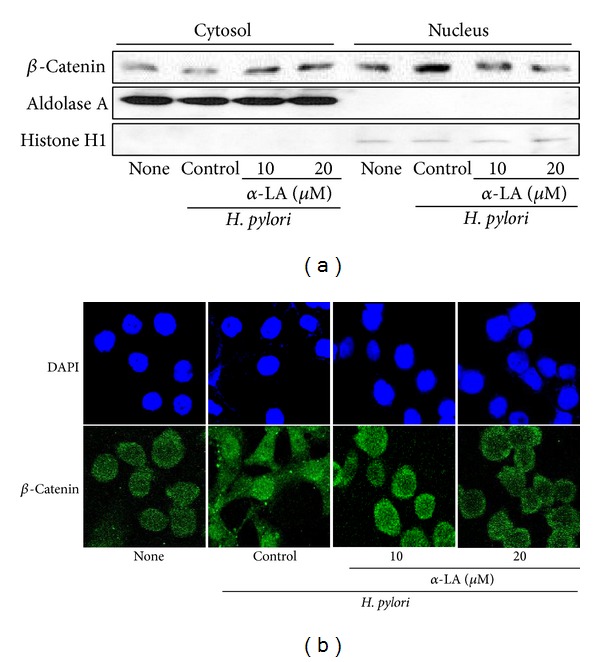
Effect of *α*-LA on activation of *β*-catenin in* H. pylori*-infected AGS cells. AGS cells were pretreated with *α*-LA for 2 h and cultured in the presence or absence of* H. pylori* for 24 h. (a) Protein levels of *β*-catenin in cytosolic and nuclear extracts were determined by Western blot analysis. Aldolase A and histone H1 were used as indices for cytosolic and nuclear extracts, respectively. (b) Immunofluorescence staining was performed to determine the levels of *β*-catenin in nucleus and cytosol. *β*-catenin was visualized using fluorescein isothiocyanate-conjugated anti-mouse IgG antibody (lower panel) with DAPI counter staining (upper panel) of the same field.

To determine the effect of *α*-LA on activation of *β*-catenin, we observed both cytosolic and nuclear levels of *β*-catenin in* H. pylori*-infected cells cultured in the presence or absence of *α*-LA (Figure 6(a)). Nuclear level of *β*-catenin was increased, but cytosolic level of *β*-catenin was decreased in* H. pylori*-infected cells. Aldolase A and histone H1, as indices for cytosol and nucleus, were not changed by* H. pylori* infection.* H. pylori-*induced activation of *β*-catenin was suppressed by treatment of *α*-LA in a dose-dependent manner. To confirm the inhibitory effect of *α*-LA on activation of *β*-catenin in* H. pylori-*infected AGS cells, nuclear localization of *β*-catenin was determined by immunofluorescence staining of *β*-catenin (Figure 6(b)).* H. pylori* induced translocation of *β*-catenin from cytosol to nucleus, which was inhibited by treatment of *α*-LA in AGS cells.

### 3.4. DPI Inhibits* H. pylori*-Induced Increase in ROS Levels, Hyperproliferation, Expression of Oncogenes, and Activation of NF-*κ*B and AP-1 in AGS Cells

**Figure 7:**
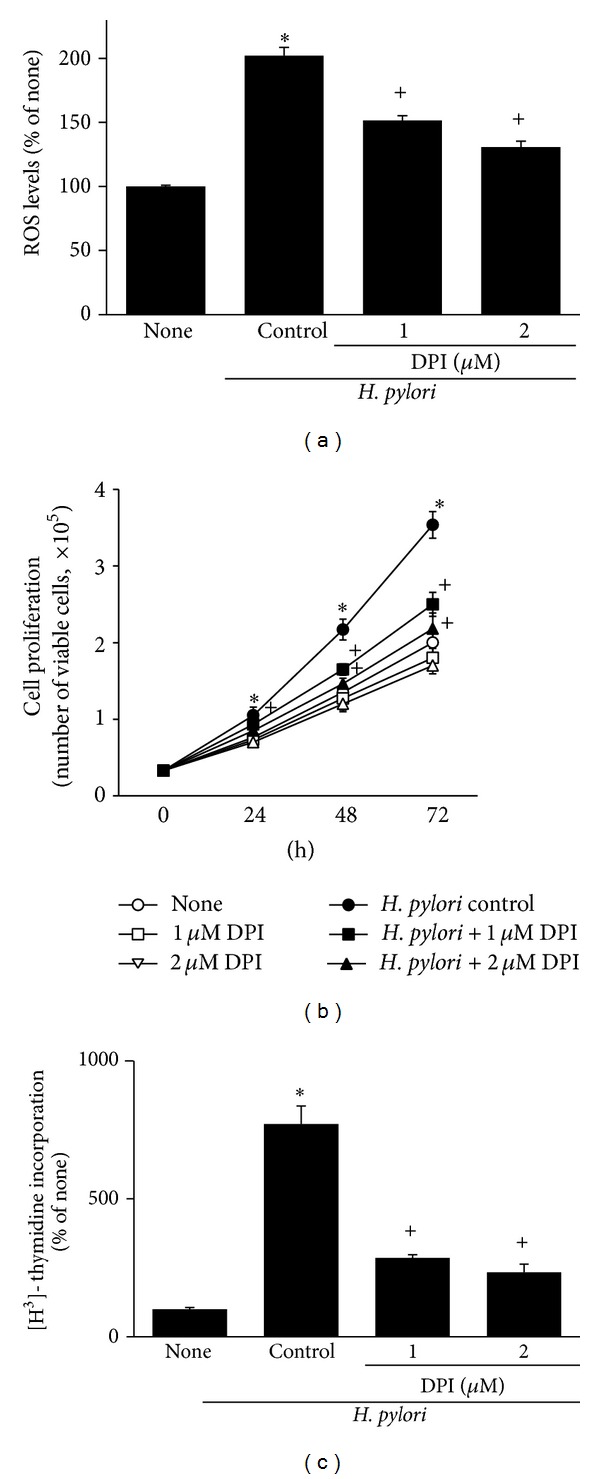
Effect of DPI on ROS levels and cell proliferation of* H. pylori-*infected AGS cells. The cells were pretreated with DPI for 2 h and cultured in the presence or absence of* H. pylori.* (a) ROS levels were determined by DCF fluorescence after 30 min of* H. pylori* infection. The levels of ROS trapped in the cells treated without DPI and cultured in the absence of* H. pylori* are considered as 100%. (b) Viable cell numbers were determined by trypan blue exclusion assay for the indicated time period. (c) The cells were treated with [^3^H] thymidine at 24 h after* H. pylori* infection and incubated for 8 h. The amount of [^3^H] thymidine incorporation of AGS cells cultured in the absence of* H. pylori* is considered as 100%. _ _*P < 0.05 versus corresponding none (the cells cultured in the absence of* H. pylori*); ^+^ *P* < 0.05 versus corresponding* H. pylori* control (the cells cultured in the presence of* H. pylori* and treated without DPI).

**Figure 8:**
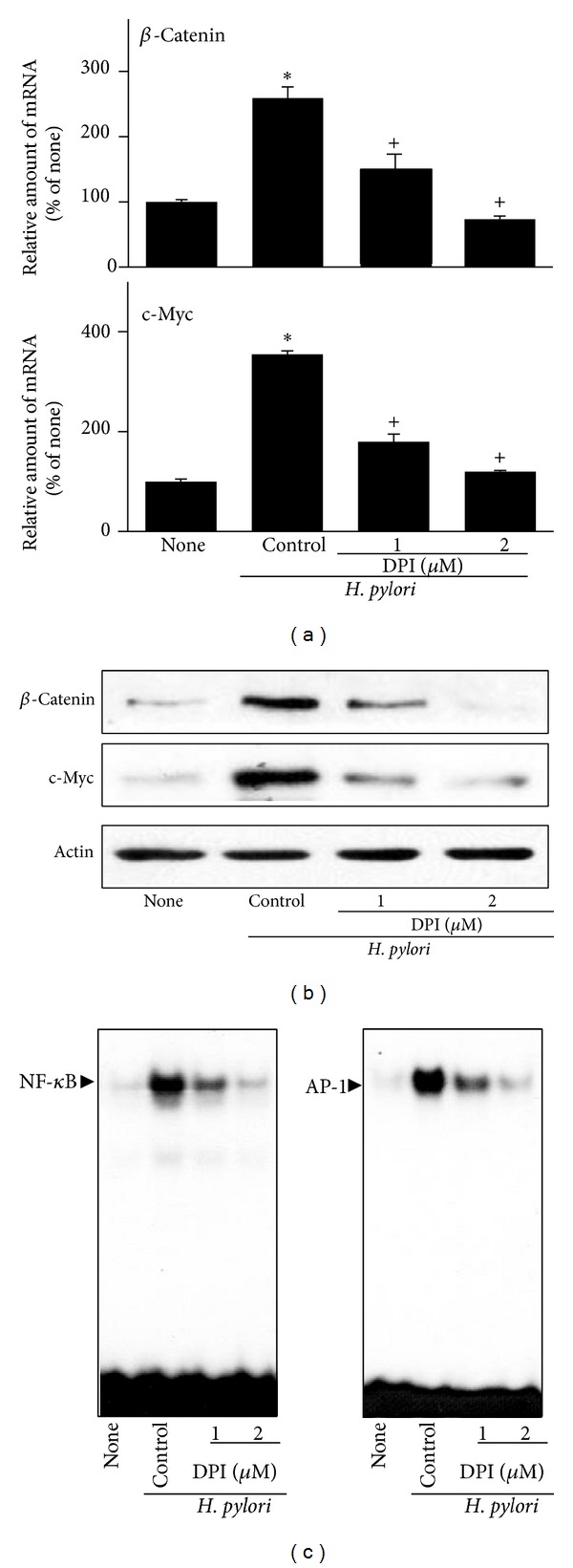
Effect of DPI on expression of *β*-catenin and c-myc as well as activation of NF-*κ*B and AP-1 in* H. pylori-*infected AGS cells. The cells were pretreated with DPI for 2 h and cultured in the presence or absence of* H. pylori *for 24 h (mRNA, protein levels) or 1 h (activation of NF-*κ*B and AP-1). (a) mRNA expression of *β*-catenin and c-myc was measured by real-time PCR analysis. (b) Protein levels of *β*-catenin and c-myc were determined by Western blot analysis. Actin served as a loading control. (c), EMSA was performed for DNA binding activities of NF-*κ*B and AP-1. _ _*P < 0.05 versus corresponding none (the cells cultured in the absence of* H. pylori*); ^+^ *P* < 0.05 versus corresponding* H. pylori* control (the cells cultured in the presence of* H. pylori* and treated without DPI).

As shown in Figure 7(a),* H. pylori*-induced increase in ROS levels was reduced by DPI treatment. DPI inhibited* H. pylori*-induced cell proliferation (determined by viable cell numbers) and DNA synthesis in AGS cells (Figures 7(b) and 7(c)). DPI itself did not affect cell viability during 72 h culture (Figure 7(b)). DPI inhibited* H. pylori*-induced expressions of *β*-catenin and c-myc in AGS cells at 24 h culture (Figures 8(a) and 8(b)). In addition, DPI showed an inhibitory effect on* H. pylori*-induced activation of NF-*κ*B and AP-1 at 1 h culture (Figure 8(c)). Inhibitory effect of DPI on* H. pylori*-induced ROS production, hyperproliferation, oncogene expression, and activation of NF-*κ*B and AP-1 was higher at 2 *μ*M than 1 *μ*M of DPI.

## 4. Discussion

The present study demonstrates that* H. pylori*-induced hyperproliferation and expression of oncogenes (*β*-catenin, c-myc) are mediated with NADPH oxidase-generated ROS and activation of redox-sensitive transcription factors (NF-*κ*B, AP-1) in* H. pylori*-infected cells. Evidence for NADPH oxidase generation of ROS during cell proliferation came from the current findings that the cell proliferation and oncogene expression were decreased by an NADPH oxidase inhibitor DPI in* H. pylori*-infected AGS cells. Since *α*-LA suppresses NADPH oxidase activation and ROS production in* H. pylori*-infected gastric epithelial cells, *α*-LA may prevent early gastric carcinogenesis associated with* H. pylori* infection, by inhibiting activation of NF-*κ*B and AP-1, expression of *β*-catenin and c-myc, and hyperproliferation of gastric epithelial cells.

Present result is supported by the previous study showing that *α*-LA attenuated ROS production and NADPH oxidase activities in the kidneys of diabetic rats [34]. Therefore, inhibition of NADPH oxidase activation may contribute to beneficial effect of *α*-LA for treatment of oxidative stress-mediated diseases including* H. pylori*-associated gastric cancer.

*α*-LA serves as an essential cofactor for mitochondrial enzymes involved in metabolism and energy production [30]. *α*-LA is a powerful antioxidant that quenches various intracellular ROS [35]. Thus, *α*-LA has been introduced to prevent or treat oxidative stress-associated diseases, such as diabetes [36, 37], ischemia-reperfusion injury [38, 39], fibrosis [40, 41], and neurodegenerative processes [42, 43].

In regard to anticancer effect of *α*-LA, *α*-LA prevented p53 degradation in colon cancer cells by inhibiting NF-*κ*B activation [44]. Anticancer effect of *α*-LA on non-small cell lung cancer cells was associated with an inhibition in the cell-cycle transition from the G1 phase to the S phase without inducing apoptosis [45]. In addition, *α*-LA inhibited migration and invasion by downregulation of cell surface *β*1-integrin expression in bladder cancer cells [46]. Present findings demonstrate that anticancer mechanism of *α*-LA is inhibition of NADPH oxidase which is upstream signaling for activation of redox-sensitive transcription factors, expression of oncogenes, nuclear translocation of *β*-catenin, and, finally, hyperproliferation of* H. pylori*-infected gastric epithelial cells.

Bandapalli et al. [47] reported that overexpression of *β*-catenin increases nuclear level of *β*-catenin and carcinogenesis including metastasis. As mentioned previously, *β*-catenin expression may be regulated by NF-*κ*B and AP-1 [21, 22]. Therefore,* H. pylori* infection may induce expression and activation of *β*-catenin by activating NF-*κ*B and AP-1 in gastric epithelial cells.

*H. pylori* strains that express the cagA and vacA genes are associated with development of chronic gastritis and intestinal metaplasia as well as increased risk for gastric cancer [48, 49].* H. pylori* has shown that approximately 50–60% of strains have a 40 kb DNA segment called the cytotoxin-associated gene (cagA) pathogenecity island (PAI) [50]. Some of the proteins encoded by cagA PAI genes are responsible for oxidant-sensitive transcription factor NF-*κ*B in gastric epithelial cells [51], which may contribute to the development of peptic ulceration, atrophic gastritis, and gastric carcinoma [52, 53]. Intestinal type gastric carcinoma is associated with high expression of c-myc through NF-*κ*B/p65 activated by* H. Pylori* cagA [54]* H. pylori* cagA mediates mitogenic signal through Src homology 2 (SH2) domain-containing protein-tyrosine phosphatase-2 (SHP-2) activation in the infected cells [55]. In addition,* H. pylori* vacA upregulates chemokine expression in human eosinophils via Ca^2+^ influx, mitochondrial ROS, and NF-*κ*B activation [56].* H. pylori* used in the study has virulence-associated genes such as vacA and cagA [57, 58]. There have been no studies on the direct effect of *α*-LA on virulence factors cagA and vacA. The present findings suggest that both cagA and vacA may contribute to the activation of NADPH oxidase, hyperproliferation, and oncogene expression in* H. pylori*-infected cells.

In the present study, *α*-LA suppressed* H. pylori*-induced activation of NADPH oxidase, ROS production, and redox-sensitive transcription factors NF-*κ*B and AP-1 in AGS cells. Since we found that expression of *β*-catenin and c-myc is regulated by NF-*κ*B and AP-1, inhibitory effect of *α*-LA on expression of *β*-catenin and c-myc and hyperproliferation may be related to suppression of NF-*κ*B and AP-1 in the infected cells.* H. pylori*-induced nuclear localization of *β*-catenin may induce expression of c-myc since *β*-catenin is reported to be shuttled into the nucleus and activate the transcription of target gene c-myc. Conclusively, *α*-LA inhibits* H. pylori*-induced hyperproliferation since c-myc acts as an oncogenic transcription factor for target genes to stimulate uncontrolled cell proliferation.

## 5. Conclusion

*α*-LA inhibits NADPH oxidase and, thus, suppresses ROS production which prevents oncogene expression, nuclear translocation of *β*-catenin, and hyperproliferation by regulating the activation of NF-*κ*B and AP-1. *α*-LA may be beneficial for prevention or therapeutic intervention for gastric carcinogenesis associated with* H. pylori *infection.
